# Tocilizumab as monotherapy or combination therapy for treating active rheumatoid arthritis: a meta-analysis of efficacy and safety reported in randomized controlled trials

**DOI:** 10.1186/s13075-016-1108-9

**Published:** 2016-09-22

**Authors:** Xavier M. Teitsma, Anne Karien A. Marijnissen, Johannes W. J. Bijlsma, Floris P. J. Lafeber, Johannes W. G. Jacobs

**Affiliations:** Department of Rheumatology and Clinical Immunology, University Medical Center Utrecht, Heidelberglaan 100, Utrecht, 3584 CX Netherlands

**Keywords:** Rheumatoid arthritis, Tocilizumab, Interleukin-6, Disease-modifying anti-rheumatic drugs, Biological

## Abstract

**Background:**

Previous studies in patients with rheumatoid arthritis (RA) have shown that switching to tocilizumab (TCZ) monotherapy (TCZ_MONO_) or combination therapy (TCZ_COMBI_) with conventional synthetic disease-modifying anti-rheumatic drugs (csDMARDs) is efficacious in reducing disease activity in patients with inadequate response to csDMARDs. However, hitherto there is no consensus on whether TCZ_MONO_ is as effective as TCZ_COMBI_. The objective of this study was therefore to evaluate the efficacy and safety of TCZ_MONO_ versus add-on TCZ_COMBI_ and both TCZ therapies versus continuing the current csDMARD therapy, by performing a systematic review and meta-analyses.

**Method:**

The MEDLINE, EMBASE and CENTRAL databases were searched until February 2016 for relevant randomized controlled trials (RCTs). We performed meta-analyses of Disease Activity Score in 28 joints (DAS28 < 2.6), American College of Rheumatology (ACR) 20/50/70 responses, adverse events (AEs) and serious AEs (SAEs) to compare the three different strategies, whereas a random-effect model was used for pooling relative risks (RR) and 95 % confidence intervals (CI). In addition, sensitivity analyses were performed for evaluating differences in study duration.

**Results:**

In total, 13 RCTs were included in the meta-analysis, involving 6679 patients. When comparing both TCZ strategies, a marginally greater proportion of patients achieving DAS28 < 2.6 (RR 1.21; 95 % CI 1.09, 1.36) and ACR50 response (RR 1.14; 95 % CI 1.03, 1.26) was found in favor of the TCZ_COMBI_ strategy. However, the risk of SAEs was also significantly higher using this strategy (RR 1.40; 95 % CI 1.03, 1.92, *p* = 0.03). Pooled effect estimates showed statistical superiority of switching to either TCZ strategy compared to continuing csDMARD therapy.

**Conclusions:**

In the management of active RA, almost similar efficacy can be expected in patients unable to tolerate csDMARDs, who switch to TCZ_MONO_ compared to inadequate responders switching to add-on TCZ_COMBI_. Although TCZ_COMBI_ is marginally superior to TCZ_MONO_ in achieving DAS28 < 2.6 and ACR50 response, this is at the cost of an increased risk of SAEs.

**Electronic supplementary material:**

The online version of this article (doi:10.1186/s13075-016-1108-9) contains supplementary material, which is available to authorized users.

## Background

In the management of rheumatoid arthritis (RA), conventional synthetic disease-modifying anti-rheumatic drugs (csDMARDs) are recommended as first-line treatment in DMARD-naïve patients for achieving remission [1–3]. Patients who have inadequate response to csDMARDs as defined by not achieving the treatment target should receive add-on therapy with a biological DMARD (bDMARD) if they have factors linked to poor prognosis (e.g., early joint damage or seropositivity). However, data obtained from large cohort studies reveal that approximately one-third of patients with RA discontinue all csDMARDs and initiate bDMARD monotherapy, mainly because of intolerance or noncompliance in taking the csDMARD [4–6]. Adverse events (AEs) are the main reason for withdrawal (>70 % of patients), with gastrointestinal symptoms being most frequently observed (>30 % of patients) [6, 7].

For patients in whom continuing csDMARD therapy is not feasible, it is important to know the effectiveness and safety of switching to bDMARD monotherapy. To this day, there is no clear preference as to which biologic agent (e.g., tumor necrosis factor inhibitors (infliximab, etanercept, adalimumab, certolizumab pegol, golimumab), rituximab (targeting B-cells), abatacept (targeting T cells) or tocilizumab (TCZ) (inhibiting interleukin 6 signaling [8]) is preferable if monotherapy must be initiated, because of a lack of head-to-head comparisons. In ADACTA, [9] a randomized, double-blind, controlled study, TCZ was shown to be superior to adalimumab in patients requiring monotherapy and could thus potentially be suitable as first-line biologic therapy. To our knowledge, this is hitherto the only bDMARD monotherapy superiority study reported. Several randomized phase III studies have shown that switching to TCZ monotherapy or combination therapy is efficacious in achieving rapid and sustained improvements within patients who do not achieve the treatment aim with csDMARD therapy [10–20]. However, there is no consensus as to whether TCZ as monotherapy is as effective as TCZ combined with csDMARDs. Although several studies have compared switching to TCZ monotherapy with add-on combination therapy, this has not yet been analyzed in meta-analyses. The objective of this study was to evaluate the efficacy and safety in patients with RA of TCZ monotherapy versus add-on TCZ combination therapy, and both TCZ therapies versus continuing the current csDMARD therapy, by performing a systematic review and meta-analyses.

## Methods

### Systematic literature search and study selection

A systematic review of the literature was conducted according to the Preferred Reporting Items for Systematic Reviews and Meta-analyses (PRISMA) statement protocol [21]. Relevant publications were identified using MEDLINE, EMBASE and Cochrane Central Register of Controlled Trials (CENTRAL). The following key search medical subject heading (MeSH) terms were selected with the help of a librarian: “Rheumatoid Arthritis”[MeSH] OR “Rheumatoid Arthritis”[tiab] AND (“Tocilizumab”[Suppl. Concept] OR “Tocilizumab”[tiab] OR “Interleukin-6”[MeSH] OR “Interleukin-6”[tiab] OR “IL-6”[tiab] OR “IL-6 receptor inhibitor”[tiab] OR “MRA”[tiab]) AND (“Randomized controlled trial”[MeSH] OR “randomized controlled trial”[tiab] OR “Clinical trial”[MeSH] OR “Clinical trial”[tiab] OR “RCT”[tiab]).

The full search strategy can be found in Additional file 1. All titles and abstracts were independently screened by two review authors (XMT and ACAM) and studies were included if: (1) they were RCTs or quasi-RCTs comparing TCZ 8 mg/kg (TCZ_MONO_) versus TCZ 8 mg/kg + csDMARD (TCZ_COMBI_), TCZ_MONO_ versus csDMARD or TCZ_COMBI_ versus csDMARD; (2) patients met the 1987 American College of Rheumatology (ACR) or 2010 ACR/European League against Rheumatism (EULAR) RA classification criteria [22, 23] and if they (3) reported ACR 20/50/70 responses, Disease Activity Score in 28 joints (DAS28), incidence of AEs and serious AEs (SAEs) within ≤52 weeks. Studies were excluded if: (1) TCZ was compared to another bDMARD; (2) only DMARD-naïve patients were included; (3) study participants were younger than 18 years; and (4) articles had not been published in the English language. If needed, the full text of the article was obtained for further assessment of eligibility. In addition, references from relevant articles were also reviewed for eligible citations. Articles not available were requested from the authors.

### Data extraction and outcome assessment

We abstracted the following data from each study that was included: design, duration, number of enrolled patients and baseline characteristics (age, gender, symptom duration, previous csDMARD and bDMARD use, erythrocyte sedimentation rate (ESR), C-reactive protein (CRP), DAS28 and Health Assessment Questionnaire score (HAQ). Outcome measurements of DAS28 remission (<2.6), ACR 20/50/70 responses, AEs and SAEs were assessed in meta-analyses. The methodological quality of the studies was independently evaluated by two review authors (XMT and ACAM) using the Cochrane Collaboration recommendations for assessing risk of bias [24]. Information was gathered and assessed on the use of random sequence generation, allocation concealment, blinding (of participants, personnel and outcome assessors), incomplete outcome data, selective reporting and sources of potential bias. Discrepancies between the two review authors were resolved by consensus with a third reviewer (JWGJ).

### Statistical analysis

Meta-analyses were performed for the following treatment-control combinations: (1) TCZ_MONO_ versus TCZ_COMBI_; (2) TCZ_MONO_ versus csDMARD; and (3) TCZ_COMBI_ versus csDMARD. Efficacy and safety measures were modeled as binary outcomes and we used a random-effects model by employing the Mantel-Haenszel method for pooling relative risks (RR) and 95 % confidence intervals (CI). During the study selection we noticed large heterogeneity between studies; hence a random-effects model was applied [25]. To analyze the sensitivity of our meta-analyses, we performed sensitivity analyses by excluding studies not reporting outcome measures at week 24, to evaluate if differences in effect sizes between studies occurred due to the variability in study duration. The *I* squared statistic (*I*^2^) was calculated to quantify heterogeneity between studies. Furthermore, publication bias was assessed by visual inspection of asymmetry in funnel plots, and relative risks were plotted on a logarithmic scale [26]. *P* values <0.05 were considered statistically significant. Statistical analyses were performed with Review Manager version 5.3 (Cochrane Collaboration, Oxford, UK).

## Results

### Literature search and study characteristics

**Fig. 1:**
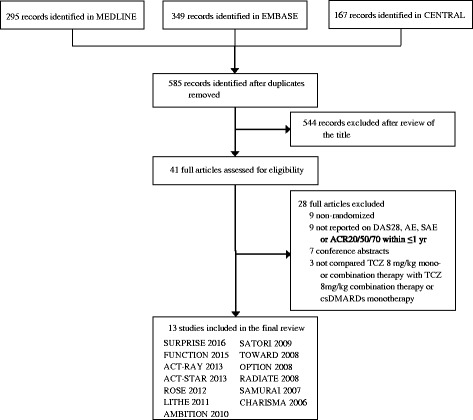
Preferred Reporting Items for Systematic Reviews and Meta-analyses flow diagram of studies included in the review. *ACR* American College of Rheumatology, *DAS28* Disease Activity Score in 28 joints, *AE* adverse event, *SAE* serious adverse event, *TCZ* tocilizumab, *csDMARDs* disease-modifying anti-rheumatic drugs

The first search was performed in May 2015 and after removing duplicates we retrieved 583 studies, of which 39 full articles were assessed for eligibility (Fig. 1). Of those studies, 11 fulfilled the inclusion criteria: Maini et al. 2006 (CHARISMA) [10], Nishimoto et al. 2007 (SAMURAI) [11], Emery et al. 2008 (RADIATE) [12], Smolen et al. 2008 (OPTION) [14], Genovese et al. 2008 (TOWARD) [13], Nishimoto et al. 2009 (SATORI) [15], Jones et al. 2010 (AMBITION) [16], Kremer et al. 2011 (LITHE) [17], Yazici et al. 2012 (ROSE) [18], Weinblatt et al. 2013 (ACT-STAR) [20] and Dougados et al. 2013 (ACT-RAY) [19]. The search was updated in February 2016 and yielded two additional studies fulfilling the inclusion criteria: Burmester et al. 2015 (FUNCTION) [27] and Kaneko et al. 2016 (SURPRISE) [28].

Patient demographics and baseline characteristics are summarized in Table 1. In total 6679 patients were included in the meta-analysis (1298 patients treated with TCZ_MONO_; 3077 patients treated with TCZ_COMBI_ and 2204 patients treated with csDMARD therapy). Demographic characteristics were comparable between studies with respect to age, gender, ESR, CRP and DAS28. Average symptom duration ranged from 4 to 14 years, except in three studies [10, 11, 27] of patients with early RA (of duration ≤2 years) only. Furthermore, eight studies [12–16, 18–20] reported 24-week results for efficacy and safety outcomes, four studies [11, 17, 27, 28] reported 52-week results and one study [10] reported outcomes at week 16. For conducting our meta-analyses, we addressed the corresponding authors of these studies and F Hoffmann-La Roche, manufacturer of TCZ and partial owner of the data, to obtain numerical data on the outcome measurements at week 24. For the 52-week studies we were able to obtain the 24-week data on efficacy outcomes; safety assessments were not available (except for the LITHE [17]).

**Table 1:** Study design and baseline characteristics of study participants presented per treatment-control combination

Study	Treatment arms	Design	Study length (weeks)	Enrolled patients (*n*)	csDMARD-naïve at baseline (yes, no)	Previous biologic therapy (%)	Mean age (years)	Female (%)	Symptom duration (years)	ESR (mm/h)	CRP (mg/l)	DAS28	HAQ
TCZ_COMBI_ vs. TCZ_MONO_													
SURPRISE (2016)	TCZ_COMBI_	Open label	52	115	No	0	56 (12)	87	4 (3)	41 (28)	12 (15)	5.1 (1)	1.0 (0.7)
	TCZ_MONO_			111			56 (3)	87	4 (3)	45 (30)	18 (26)	5.3 (1.2)	1.0 (0.7)
FUNCTION (2015)	TCZ_COMBI_	Double-blind	52	291	No^f^	0	50 (14)	79	6 (6)^g^	53 (30)	26 (30)	6.7 (1.1)	1.5 (0.6)
	TCZ_MONO_			292			50 (12)	75	6 (6)^g^	51 (28)	25 (32)	6.7(1.0)	1.6 (0.7)
ACT-RAY (2013)	TCZ_COMBI_	Open-label, double-blind^c^	24	277	No	0	53 (13)	82	8 (8)	NR	NR	6.3 (1)	1.5 (0.7)
	TCZ_MONO_			276			54 (12)	79	8 (8)			6.4 (1)	1.5 (0.6)
ACT-STAR (2013)^a^	TCZ_COMBI_	Open-label	24	360	No	67	54 (12)	78	11 (9)	NR	14 (21)	5.5 (1)	NR
	TCZ_MONO_			163		87	54 (13)	80	14 (10)		19 (33)	6.0 (1)	
CHARISMA (2006)^b^	TCZ_COMBI_	Double-blind	16	50	No	14	50 (NR)	78	11 (NR)^c^	39 (NR)	24 (NR)	6.5 (NR)	NR
	TCZ_MONO_			52			50 (NR)	73	9 (NR)^c^	39 (NR)	22 (NR)	6.4 (NR)	
TCZ_COMBI_ vs. csDMARD													
FUNCTION (2015)	TCZ_COMBI_	Double-blind	52	291	No	0	50 (14)	79	6 (6)^c^	53 (30)	26 (30)	6.7 (1.1)	1.5 (0.6)
	csDMARD			289			50 (13)	80	5 (6)^c^	50 (27)	23 (27)	6.6 (1.0)	1.5 (0.7)
ROSE (2012)	TCZ_COMBI_	Double-blind	24	409	No	38	55 (12)	80	9 (9)	46 (24)	17 (21)	6.5 (1)	4.1 (1.7)^h^
	csDMARD			205		38	56 (12)	84	9 (9)	47 (22)	17 (22)	6.6 (1)	4.0 (2.1)^h^
LITHE (2011)^a^	TCZ_COMBI_	Double-blind^d^	52	398	No	11	53 (12)	82	9 (NR)	46 (25)	23 (26)	6.6 (1)	1.5 (0.6)
	csDMARD			393		12	51 (12)	83	9 (NR)	47 (25)	22 (25)	6.5 (1)	1.5 (0.6)
TOWARD (2008)	TCZ_COMBI_	Double-blind	24	803	No	NS	53 (13)	81	10 (9)	48 (28)	26 (32)	6.7 (1)	1.5 (0.6)
	csDMARD			413			54 (13)	84	10 (9)	49 (28)	26 (47)	6.6 (1)	1.5 (0.6)
OPTION (2008)^a^	TCZ_COMBI_	Double-blind	24	205	No	5	51 (12)	85	8 (7)	51 (27)	26 (26)	6.8 (1)	1.6 (0.6)
	csDMARD			204		9	51 (12)	78	8 (7)	50 (26)	24 (28)	6.8 (1)	1.5 (0.6)
RADIATE (2008)^a^	TCZ_COMBI_	Double-blind	24	170	No	100	54 (13)	84	13 (9)	49 (28)	28 (33)	6.8 (1)	1.7 (0.6)
	csDMARD			158			53 (13)	79	11 (9)	55 (33)	37 (41)	6.8 (1)	1.7 (0.6)
CHARISMA (2006)^b^	TCZ_COMBI_	Double-blind	16	50	No	14	50 (NR)	78	11 (NR)^c^	39 (NR)	24 (NR)	6.5 (NR)	NR
	csDMARD			49			51 (NR)	78	11 (NR)^c^	43 (NR)	32 (NR)	6.8 (NR)	
TCZ_MONO_ vs. csDMARD													
FUNCTION (2015)	TCZ_MONO_	Double-blind	52	292	No	0	50 (12)	75	6 (6)^c^	51 (28)	25 (32)	6.7(1.0)	1.6 (0.7)
	csDMARD			289			50 (13)	80	5 (6)^c^	50 (27)	23 (27)	6.6 (1.0)	1.5 (0.7)
AMBITION (2010)	TCZ_MONO_	Double-blind	24	286	No	8	51 (13)	83	6 (8)	50 (28)	30 (33)	6.8 (1)	1.6 (0.7)
	csDMARD			284		7	50 (13)	79	6 (8)	49 (26)	31 (34)	6.8 (1)	1.5 (0.6)
SATORI (2009)	TCZ_MONO_	Double-blind	24	61	No	NS	53 (11)	90	9 (8)	52 (28)	30 (20)	6.1 (1)	NR
	csDMARD			64			51 (12)	75	9 (7)	52 (24)	32 (26)	6.2 (1)	
SAMURAI (2007)	TCZ_MONO_	Open-label^e^	52	157	No	NS	53 (12)	80	2 (1)	71 (28)	47 (29)	6.5 (1)	NR
	csDMARD			145			53 (13)	82	2 (1)	71 (25)	49 (29)	6.4 (1)	
CHARISMA (2006)^b^	TCZ_MONO_	Double-blind	16	52	No	14	50 (NR)	73	9 (NR)^c^	39 (NR)	22 (NR)	6.4 (NR)	NR
	csDMARD			49			51 (NR)	78	11 (NR)^c^	43 (NR)	32 (NR)	6.8 (NR)	

Values are expressed as mean (standard deviation) unless otherwise indicated. ^a^Tocilizumab (TCZ) 4 mg + conventional synthetic disease-modifying anti-rheumatic drug (csDMARD) comparator group is excluded in this overview; ^b^TCZ 2 mg, TCZ 4 mg, TCZ 2 mg + csDMARD and TCZ 4 mg + csDMARD comparator groups were excluded; ^c^TCZ was given open-label, treatment with methotrexate (MTX) was double-blind; ^d^first-year therapy was double-blind followed by a second year of open-label therapy; ^e^open-label for clinical efficacy endpoints, single-blind only for radiographic evaluation; ^f^all patients were MTX-naïve, but only approximately 80 % were entirely csDMARD-naïve; ^g^months; ^h^Health Assessment Questionnaire-physical function (HAQ-PF) score. *TCZ* tocilizumab, *ESR* erythrocyte sedimentation rate, *CRP* C-reactive protein, *DAS28* Disease Activity Score in 28 joints, *NS* percentage not specified, *NR* not reported

Most studies included [10, 12, 14–17, 19, 27, 28] used methotrexate (MTX) as the csDMARD and folic acid (≥5 mg/week) was given to all patients to minimize MTX-related toxicity, except in two studies [15, 19] in which only 51–81 % of the patients received folic acid. Folate supplementation was not reported in two other studies [27, 28]. Before study entry, all patients were on stable doses of MTX or other csDMARDs for ≥4 weeks before switching to the TCZ_MONO_ or TCZ_COMBI_ strategy, except in the FUNCTION [27] study in which the majority (81 %) of patients were DMARD-naïve, and the ACT-STAR [20] study in which patients were on bDMARD monotherapy before switching to the TCZ_MONO_ strategy. There were also differences between studies in prior anti-tumor necrosis factor alpha (aTNFα) treatment. In four studies [10, 14, 16, 17], only a small proportion (5-14 %) of the patients had received aTNFα medication prior to inclusion, in contrast to other studies [12, 18, 20] in which 38–100 % of patients had received aTNFα. In several studies [11, 13, 15], aTNFα treatment was allowed before the start of the study; however, this proportion was not specified and in the ACT-RAY [19], FUNCTION [27] and SURPRISE [28] studies, patients were excluded if they had previously received aTNFα treatment. In the studies in which prior aTNFα treatment was allowed, washout periods were applied before study entry to reduce treatment effects, except in the ACT-STAR study [20].

The most commonly reported AEs in the studies included in our meta-analyses were infections (e.g., skin and respiratory infections), skin disorders (e.g., rash) and gastrointestinal symptoms (e.g., nausea). The studies reported an incidence of AEs and SAEs in patients treated with TCZ_MONO_ ranging from 59 to 92 % and from 4 to 18 %, respectively [10, 11, 15, 16, 19, 20]. For TCZ_COMBI_, these ranges were 54 to 84 % and 6 to14%, respectively, [10, 12–14, 18–20] and for csDMARD therapy 47 to 82 % and 3 to 13 %, respectively [10–16, 18].

### Efficacy outcomes

#### DAS28 < 2.6

**Fig. 2:**
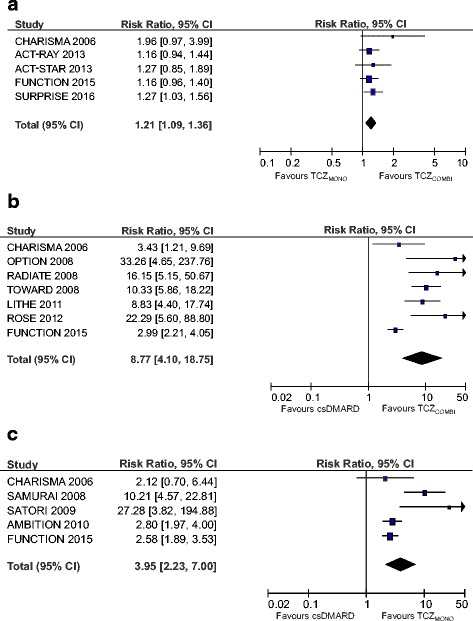
Forest plots of Disease Activity Score remission (<2.6) assessed in 28 joints (DAS28) comparing tocilizumab combination therapy (*TCZ*_*COMBI*_) with tocilizumab monotherapy (*TCZ*_*MONO*_) (**a**), TCZ_COMBI_ with a conventional synthetic disease-modifying anti-rheumatic drug (*csDMARD*) (**b**) and TCZ_MONO_ with a csDMARD (**c**)

Forest plots of DAS28 are shown in Fig. 2. In the TCZ monotherapy and combination strategy, pooled effect estimates for achieving remission were significantly higher (RR 3.95; 95 % CI 2.23, 7.00, *p* < 0.001 and RR 8.77; 95 % CI 4.10, 18.75, *p* < 0.001, respectively) compared to csDMARD therapy. On comparison of the two TCZ strategies, the effect estimate was significantly higher (RR 1.21; 95 % CI 1.09, 1.36, *p* < 0.001) for the combination strategy.

#### ACR20 response

Forest plots of the ACR responses are shown in Additional file 2. Pooled effect estimates for achieving ACR20 response were significantly higher for both the TCZ_MONO_ (RR 1.68; 95 % CI 1.21, 2.32, *p* = 0.002) and TCZ_COMBI_ (RR 2.10; 95 % CI 1.48, 2.99, *p* < 0.001) strategy, when compared to csDMARD therapy. There was no difference between the two TCZ strategies (*p* = 0.11).

#### ACR50 response

The proportion of ACR50 responders was statistically higher with both TCZ strategies compared to csDMARD therapy (TCZ_MONO_: RR 1.87; 95 % CI 1.19, 2.95, *p* = 0.007 and TCZ_COMBI_: RR 3.00; 95 % CI 1.80, 4.99, *p* < 0.001). Furthermore, patients treated with the add-on TCZ_COMBI_ strategy achieved an ACR50 response significantly more often than with the TCZ_MONO_ strategy; however, this effect estimate was relatively small (RR 1.14; 95 % CI 1.03, 1.26, *p* = 0.008).

#### ACR70 response

The pooled effect estimates of ACR70 response rates were significantly higher in patients treated with the TCZ_MONO_ and TCZ_COMBI_ strategy compared to patients treated with csDMARD therapy (RR 2.11; 95 % CI 1.18, 3.78, *p* = 0.01 and RR 5.32; 95 % CI 2.31, 12.25, *p* < 0.001, respectively). There was no statistically significant difference between the two TCZ strategies (*p* = 0.14).

### Safety outcomes

#### Adverse events

**Fig. 3:**
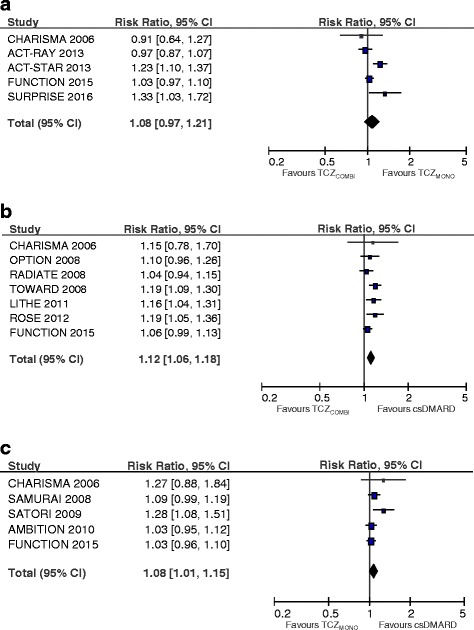
Forest plots of adverse events comparing tocilizumab combination therapy (*TCZ*_*COMBI*_) with tocilizumab monotherapy (*TCZ*_*MONO*_) (**a**), TCZ_COMBI_ with a conventional synthetic disease-modifying anti-rheumatic drug (*csDMARD*) (**b**) and TCZ_MONO_ with a csDMARD (**c**)

For both TCZ strategies, the pooled risk estimates for experiencing one or more AE during treatment was significantly higher compared to csDMARD therapy (TCZ_MONO_: RR 1.08; 95 % CI 1.01, 1.15, *p* = 0.03; TCZ_COMBI_: RR 1.12; 95 % CI 1.06, 1.18, *p* < 0.001, Fig. 3). In the meta-analyses of TCZ_MONO_ versus TCZ_COMBI_, there was no statistically significant difference between the strategies (*p* = 0.17).

**Fig. 4:**
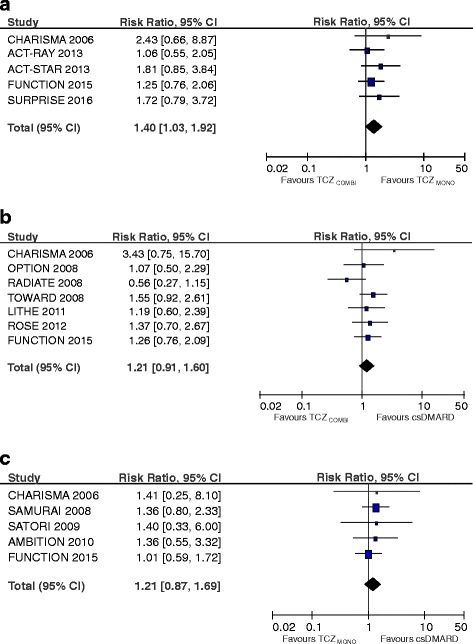
Forest plots of serious adverse events comparing tocilizumab combination therapy (*TCZ*_*COMBI*_) with tocilizumab monotherapy (*TCZ*_*MONO*_) (**a**), TCZ _COMBI_ with a conventional synthetic disease-modifying anti-rheumatic drug (*csDMARD*) (**b**) and TCZ_MONO_ with a csDMARD (**c**)

SAEs occurred more frequently in the TCZ_MONO_ (RR 1.21; 95 % CI 0.87, 1.69) and TCZ_COMBI_ (RR 1.21; 95 % CI 0.91, 1.60) strategy compared to csDMARD therapy (Fig. 4). However, this increased risk was not statistically significant for either TCZ strategy (*p* = 0.26 and *p* = 0.19, respectively). When comparing the incidence of SAEs with the TCZ_MONO_ and TCZ_COMBI_ strategies, the pooled risk estimate was significantly higher with the combination strategy (RR 1.40; 95 % CI 1.03, 1.92, *p* = 0.03).

### Publication bias

We found no clear indication of publication bias on visual inspection of funnel plots (see Additional file 3). The effect estimates of most studies were within the expected 95 % CI, indicating no clear pattern of bias. However, assessment of publication bias in such a small number of studies remains controversial and should be interpreted with caution [29].

### Sensitivity analysis

The pooled effect estimates of the sensitivity analyses are shown in Table 2; studies were excluded if they did not report 24-week results. In the efficacy and safety meta-analysis of TCZ_COMBI_ versus TCZ_MONO_, one [10] and three studies [10, 27, 28] were excluded, respectively. The effect estimates of the efficacy outcomes remained relatively unchanged; however, the pooled risk for SAEs changed from being significant (RR 1.40; 95 % CI 1.03, 1.92, *p* = 0.03) to non-significant (RR 1.34; 95 % CI 0.79, 2.27, *p* = 0.27), indicating the two TCZ strategies were equally safe. In the efficacy and safety meta-analysis of TCZ_COMBI_ versus a csDMARD, one [10] and two studies [10, 27] were excluded, respectively; the sensitivity analyses showed greater effect estimates for DAS28 < 2.6 and ACR20/50/70 responses in favor of the TCZ strategy; the risk of AEs and SAEs did not change significantly. In the meta-analysis of TCZ_MONO_ versus a csDMARD, one study [10] was excluded from analysis of the efficacy outcomes and three studies [10, 11, 27] from analysis of the safety outcomes; the pooled risk of AEs changed from being significant (RR 1.08; 95 % CI 1.01, 1.15, *p* = 0.03) to non-significant (RR 1.13; 95 % CI 0.92, 1.39, *p* = 0.24) in favor of the TCZ strategy.

**Table 2:** Efficacy and safety outcomes of tocilizumab 8 mg/kg monotherapy and combination therapy comprising tocilizumab and a conventional synthetic disease modifying anti-rheumatic drug

	Meta-analysis			Sensitivity analyses^a^		
Outcome measures	RR	95 % CI	*P*	RR	95 % CI	*P*
TCZ_COMBI_ vs. TCZ_MONO_						
DAS28 < 2.6	1.21	1.09, 1.36	<0.001	1.20	1.07, 1.34	0.002
ACR20	1.05	0.99, 1.12	0.11	1.05	0.98, 1.11	0.17
ACR50	1.14	1.03, 1.26	0.008	1.13	1.02, 1.25	0.02
ACR70	1.19	0.94, 1.51	0.14	1.12	0.95, 1.33	0.19
AEs	1.08	0.97, 1.21	0.17	1.09	0.86, 1.38	0.48
SAEs	1.40	1.03, 1.92	0.03	1.34	0.79, 2.27	0.27
TCZ_COMBI_ vs. csDMARD						
DAS28 < 2.6	8.77	4.10, 18.75	<0.001	10.39	4.38, 24.65	<0.001
ACR20	2.10	1.48, 2.99	<0.001	2.15	1.45, 3.19	<0.001
ACR50	3.00	1.80, 4.99	<0.001	3.24	1.82, 5.78	<0.001
ACR70	5.32	2.31, 12.25	<0.001	6.23	2.29, 16.93	<0.001
AEs	1.12	1.06, 1.18	<0.001	1.14	1.07, 1.20	<0.001
SAEs	1.21	0.91, 1.60	0.19	1.13	0.80, 1.60	0.48
TCZ_MONO_ vs. csDMARD						
DAS28 < 2.6	3.95	2.23, 7.00	<0.001	4.50	2.34, 8.64	<0.001
ACR20	1.68	1.21, 2.32	0.002	1.71	1.18, 2.48	0.005
ACR50	1.87	1.19, 2.95	0.007	2.01	1.18, 3.42	0.01
ACR70	2.11	1.18, 3.78	0.01	2.49	1.29, 4.81	0.007
AEs	1.08	1.01, 1.15	0.03	1.13	0.92, 1.39	0.24
SAEs	1.21	0.87, 1.69	0.26	1.37	0.64, 2.93	0.42

^a^The CHARISMA study was excluded from all meta-analyses; the FUNCTION study was excluded from all meta-analyses of safety outcomes (adverse events (AEs) and serious AEs (SAEs)); the SAMURAI study was excluded from meta-analyses of the safety of tocilizumab monotherapy (TCZ_MONO_) vs. a conventional synthetic disease-modifying anti-rheumatic drug (csDMARD); the SURPRISE study was excluded (from meta-analyses of the safety of tocilizumab combination therapy (TCZ_COMBI_) vs. TCZ_MONO._ *RR* relative risk, *CI* confidence interval, *DAS28* Disease Activity Score in 28 joints, *ACR* American college of Rheumatology, *AEs* adverse events, *SAEs* serious AEs

### Assessment of heterogeneity

A detailed description of the heterogeneity per meta-analysis is presented in Additional file 4. Heterogeneity (*I*^2^) was the lowest in the TCZ_COMBI_ versus TCZ_MONO_ comparison, indicating minimal differences in effect sizes between studies. However, in the majority of the other meta-analyses we found substantial heterogeneity (>50 %). Furthermore, the results of our sensitivity analyses show that heterogeneity between studies was even slightly increased by excluding studies reporting outcome measures other than at 24 weeks, which indicates that the between-study variation in our meta-analyses was not majorly affected by the differences in study duration.

### Risk of bias assessment

The risk of bias assessment is shown in Additional file 5. To appraise the generalizability of our findings, we assessed the validity of the studies included. Generally, there was a low risk of selection bias because of adequate allocation concealment, except for the OPTION study [14]. In this study, the randomization list was provided by F Hoffmann-La Roche, the manufacturer of TCZ. The risk of performance and detection bias was also low because studies were double-blinded, except for the ACT-RAY, ACT-STAR, SURPRISE and SAMURAI studies, which were open-label [19, 20, 28] and single-blinded [11] studies, respectively. Most authors received consulting and/or speaking fees, honoraria, held a patent for TCZ, held stock or stock options with the manufacturer or were employees of F Hoffmann-La Roche. Also, except for the SURPRISE study [28], all studies were directly supported by F Hoffmann-La Roche or Chugai Pharmaceutical. Although vested interests do not necessarily lead to bias or impaired study methodology, these aspects should, however, be weighed in the balance in coming to a conclusion when determining the efficacy and safety of TCZ in patients with RA.

## Discussion

This is the first meta-analysis comparing the efficacy and safety of TCZ monotherapy versus TCZ add-on combination therapy in patients with RA. Our results show that the efficacy of TCZ_MONO_ is nearly equivalent to TCZ_COMBI_ in the management of active RA. Although the effect estimate for achieving DAS28 < 2.6 and ACR50 response was significantly higher with the TCZ_COMBI_ strategy, this is at the cost of a significant increase in the risk of SAEs when compared to TCZ_MONO_. Thus, if patients do not achieve the treatment target after initiating csDMARD therapy because of intolerance, switching to TCZ_MONO_ is a feasible option in clinical practice, whereas similar efficacy can be expected compared to inadequate responders to csDMARDs who switch to add-on TCZ_COMBI_ therapy.

TCZ is the first biologic agent to show comparable efficacy when used as monotherapy or combination therapy, whereas other biologic agents have consistently been reported to be significantly less effective when used as monotherapy [30–33]. To determine if TCZ_MONO_ is also superior to other biologic monotherapies, it is necessary to make direct comparisons in patients who require this therapy. However, the ADACTA study [9] is hitherto the only head-to-head randomized trial comparing two different biologic monotherapies (adalimumab versus TCZ), whereas TCZ was found to be superior for reducing signs and symptoms of RA in patients for whom continuation of MTX was discouraged. These findings are endorsed in a network analysis indirectly comparing biologic monotherapy trials, whereas TCZ has also been associated with greater efficacy than aTNFα [34]. However, further research is needed to determine the potential superiority of TCZ as first-line biologic therapy.

The results of our meta-analyses confirm and extend findings from previous studies. Although systematic reviews often do not directly lead to new insights, our review provides stronger evidence compared to clinical trials and also has several other advantages. Meta-analyses are able to examine rare events more adequately by accruing data from several studies and therefore increasing the likelihood of finding relevant differences. The majority of the studies included in our systematic review had samples sizes calculated on the basis of efficacy outcomes, which are frequently observed, and were thus not powered to detect significant differences in the incidence of less frequent events such as SAEs.

In this review, five studies [10, 19, 20, 27, 28] were included that compared TCZ_MONO_ with TCZ_COMBI_ and none of them demonstrated a significant difference between the two strategies in the incidence of SAEs. However, our meta-analyses showed a significantly higher risk (RR 1.40; 95 % 1.03, 1.92, *p* = 0.03) of SAEs with the TCZ_COMBI_ strategy. By pooling effect estimates, meta-analyses are better able to determine the true efficacy and safety of therapy compared to individual studies, and are therefore essential for making future recommendations on the management of RA. Apart from more adequately estimating the benefit and harms of a therapy, meta-analyses are also able to evaluate the inconsistency between studies and quantify treatment effects, and are invaluable to practitioners, as they summarize the latest evidence [35, 36].

In our meta-analysis, TCZ was only assessed at a dose of 8 mg/kg. Previous clinical trials [10, 12, 14, 17, 20, 37] consistently report superior efficacy of 8 mg/kg compared to 4 mg/kg in decreasing disease activity and preventing radiographic evidence of progression. The findings of these studies were confirmed in several systematic reviews, whereas meta-analyses have shown significantly greater efficacy with the 8-mg/kg dose [38, 39]. In another review performed by Campbell et al. [40], the risk of AEs was not significantly different between the two different doses, which supports the superior dose–response relationship of the higher dose. TCZ inhibits the binding of IL-6 to its receptor, which has its direct effects on acute phase reactants (APR) such as CRP and ESR. Although reduction in APR was sustained in patients treated with 8 mg/kg, these decreases were not maintained with 4 mg/kg, possibly indicating less sufficient targeting of the IL-6 pathway and therefore resulting in less efficacy [14, 17, 41].

In this review we also evaluated the efficacy and safety of both TCZ strategies versus csDMARD therapy. According to current guidelines, newly diagnosed patients with RA should receive csDMARD therapy as the first-line treatment, and in the case of inefficacy or intolerability, switch to or add a biologic DMARD. Before making assumptions as to whether TCZ_MONO_ or TCZ_COMBI_ would be adequate to decrease symptoms in patients not achieving the treatment target with csDMARD therapy alone, it is necessary to compare the efficacy of both these strategies with the standard of care. Although a proportion of patients did not initially achieve remission with csDMARD therapy, this does not necessary mean that this therapy was not effective at all. Thus, the efficacy of TCZ_MONO_ could be not significantly better than that of the csDMARD therapy in these patients who are more difficult to treat, and then, switching to TCZ_MONO_ would thus not be a good option for patients who do not respond to csDMARDs.

Although this is the first meta-analysis comparing TCZ_MONO_ with csDMARD therapy, TCZ_COMBI_ has already been compared to csDMARD therapy in previous meta-analyses [38–40]. Our meta-analysis of this treatment-control combination differs to these studies because: (1) several new large RCTs have been published; (2) 24-week results were obtained from studies with longer follow up; and (3) we performed sensitivity analyses to assess the heterogeneity between studies. The results of our meta-analyses show that switching to TCZ_MONO_ and TCZ_COMBI_ therapy is superior to continuing csDMARD therapy alone in patients with active RA. However, the treatment effect of TCZ is likely to be enhanced by the study design of the RCTs, because in studies with a csDMARD comparator group [10–18], patients were already on this therapy prior to inclusion, except in the FUNCTION study [27], whereas 81 % of the patients were DMARD-naïve.

TCZ, as monotherapy or combination therapy, has also shown to be effective as first-line therapy in newly diagnosed treatment-naïve patients with RA. In U-Act-Early [42], a recently published randomized, multicenter, three-parallel-arm, double-blind, treat-to-target study, DMARD-naïve patients were allocated to start on TCZ_MONO_, TCZ_COMBI_ or MTX therapy. Both TCZ strategies were found to be more effective in achieving sustained remission (TCZ_COMBI_ 86 %, TCZ_MONO_ 83 %) compared to MTX (44 %, *p* < 0.001). This study endorses the immediate initiation of TCZ in early RA, with or without MTX, when used in a treat-to-target approach, including tapering of medication when remission is achieved.

Several weaknesses are apparent in this meta-analyses, which will be addressed. First, studies included in our review were heterogeneous in respect to csDMARD therapy. The majority used MTX monotherapy [10, 12, 14–17, 19] as control therapy; however, in four studies [11, 13, 18, 20] several csDMARDs were permitted. Furthermore, MTX was given at a lower dose in Japanese studies [11, 15] compared to studies performed in Caucasian populations [10, 12, 14, 16, 17, 19] because of the different health regulations. Second, the treatment efficacy of TCZ as assessed using the DAS28 should be interpreted with caution because of the prominent effect of IL-6 inhibition by TCZ on APR, which can lead to exaggerated response rates when compared to csDMARD or other bDMARD therapies. Thus, we also used ACR response criteria for evaluating efficacy, which yielded similar findings when compared to DAS28, endorsing the superiority of switching to TCZ_MONO_ or TCZ_COMBI_ therapy compared to continuing csDMARD therapy. Third, studies also differed with respect to study duration. Sensitivity analyses were performed to assess whether studies with follow up shorter or longer than 24 weeks reported systematically different treatment effects. In general, the effect estimates and the between-study variation did not change significantly and thus, does not change our previous conclusions. However, it may indicate that other factors such as demographic characteristics (e.g., early versus established RA) and prior DMARD use (e.g., MTX-naïve versus non-naïve patients) probably contribute more to the heterogeneity. Unfortunately because of the limited number of studies included in this review, we were not able to explore these potential sources of between-study variability.

## Conclusions

In conclusion, in the management of active RA, switching to TCZ_MONO_ is a good option for patients who cannot tolerate csDMARDs, whereas similar efficacy can be expected compared to TCZ_COMBI_ therapy. Although TCZ_COMBI_ is marginally superior to TCZ_MONO_ in terms of achieving DAS28 < 2.6 and ACR50 response, this is at the cost of a significantly increased risk of SAEs. TCZ is the first biologic agent to show comparable efficacy when used as monotherapy or combination therapy.

## Additional files

Additional file 1: Detailed description of the full search strategy. (DOCX 13 kb) Additional file 2: Forest plots of American College of Rheumatology 20 (a), 50 (b), and 70 (c) responses. Meta-analyses of ACR responses for the following treatment-control combinations: (1) TCZ_MONO_ vs. TCZ_COMBI_; (2) TCZ_MONO_ vs. csDMARD; and (3) TCZ_COMBI_ vs. csDMARD. (DOCX 31 kb) Additional file 3: Funnel plots of a DAS28 < 2.6, b ACR20, c ACR50 responses, d ACR70 responses, e AEs and f SAEs. Funnel plots of efficacy and safety outcomes for the following treatment-control combinations: (1) TCZ_MONO_ vs. TCZ_COMBI_; (2) TCZ_MONO_ vs. csDMARD; and (3) TCZ_COMBI_ vs. csDMARD. (DOCX 63 kb) Additional file 4: Variance in heterogeneity per meta-analysis. Table showing the heterogeneity between studies of DAS28 < 2.6, ACR 20/50/70 responses, AEs and SAEs. (DOCX 17 kb) Additional file 5: Assessment of quality of studies (+ indicates low risk of bias, − indicates high risk of bias and ? indicates the risk of bias is unclear). Table showing the risk of bias for each study included in this meta-analyses. (DOCX 30 kb)

## Abbreviations

ACR: American College of Rheumatology
ACR20/50/70: 20 %/50 %/70 % improvement according to American College of Rheumatology criteria
AE: Adverse event
APR: Acute phase reactants
aTNFα: Anti-tumor necrosis factor alpha
bDMARD: Biological disease-modifying anti-rheumatic drug
CENTRAL: Cochrane Central Register of Controlled Trials
CI: Confidence interval
CRP: C-reactive protein
csDMARD: Conventional synthetic disease-modifying anti-rheumatic drug
DAS28: Disease Activity Score in 28 joints
DMARD: Disease-modifying anti-rheumatic drug
EMBASE: Exerpta Medica Database
ESR: Erythrocyte sedimentation rate
EULAR: European League Against Rheumatism
HAQ: Health Assessment Questionnaire
IL-6: Interleukin-6
MeSH: Medical subject headings
MTX: Methotrexate
PRISMA: Preferred Reporting Items for Systematic Reviews and Meta-analyses
RA: Rheumatoid arthritis
RCT: Randomized controlled trial
RR: Relative risk
SAE: Serious adverse event
TCZ: Tocilizumab
TCZ_COMBI_: Tocilizumab combination therapy
TCZ_MONO_: Tocilizumab monotherapy
