# Supplementary material for: Tocilizumab as monotherapy or combination therapy for treating active rheumatoid arthritis: a meta-analysis of efficacy and safety reported in randomized controlled trials
Source: Arthritis Res Ther. 2016 Sep 22;18:211. doi: 10.1186/s13075-016-1108-9 (PMC5034420; doi:10.1186/s13075-016-1108-9)
Supplement: Additional file 1: — Detailed description of the full search strategy. (DOCX 13 kb) [file 13075_2016_1108_MOESM1_ESM.docx]

Additional file 1.

***MEDLINE***

#1 Search “Arthritis, Rheumatoid”[Mesh]
#2 Search “rheumatoid arthritis”[Title/Abstract]
#3 Search “tocilizumab”[Supplementary Concept]
#4 Search “tocilizumab”[Title/Abstract]
#5 Search “Interleukin-6”[Mesh]
#6 Search “interleukin-6”[Title/Abstract]
#7 Search “IL-6”[Title/Abstract]
#8 Search “IL 6 receptor inhibitor”[Title/Abstract]
#9 Search “mra”[Title/Abstract]
#10 Search “Randomized Controlled Trial”[Publication Type]
#11 Search “randomized controlled trial”[Title/Abstract]
#12 Search “Clinical Trial”[Publication Type]
#13 Search “clinical trial”[Title/Abstract]
#14 Search "rct"[Title/Abstract]
#15 Search (#1) OR (#2)
#16 Search ((((((#3) OR #4) OR #5) OR #6) OR #7) OR #8) OR #9
#17 Search ((((#19) OR #11) OR #12) OR #13) OR #14
#18 Search ((#15) AND #16) AND #17

***EMBASE***

#1 ‘rheumatoid arthritis’/de
#2 ‘rheumatoid arthritis’:ab:ti
#3 ‘tocilizumab’/exp
#4 ‘tocilizumab’:ab:ti
#5 ‘interleukin 6’/exp
#6 ‘interleukin 6’:ab:ti
#7 il AND 6 AND (‘receptor’/exp OR receptor) AND inhibitor:ab:ti
#8 ‘mra’:ab:ti
#9 ‘randomized controlled trial’:ab:ti
#10 ‘randomized controlled trial’/exp
#11 ‘clinical trial’/exp
#12 ‘clinical trial’:ab:ti
#13 ‘rct’:ab:ti
#14 #1 OR #2
#15 #3 OR #4 OR #5 OR #6 OR #7 OR #8
#16 #9 OR #10 OR #11 OR #12 OR #13
#17 #14 AND #15 AND #16

***CENTRAL***#1 “Arthritis, Rheumatoid”[MeSH]
#2 “rheumatoid arthritis”:ti,ab,kw
#3 “Tocilizumab”[MeSH]
#4 “tocilizumab”:ti,ab,kw
#5 “interleukin 6”:ti,ab,kw
#6 “IL 6”:ti,ab,kw
#7 “IL 6 receptor inhibitor”:ti,ab,kw
#8 “mra”:ti,ab,kw
#9 “Randomized Controlled Trial”[MeSH]
#10 “randomized controlled trial”:ti,ab,kw
#11 “Clinical Trial”[MeSH]
#12 “clinical trial”:ti,ab,kw
#13 “rct”:ti,ab,kw
#14 #1 OR #2
#15 #3 OR #4 OR #5 OR #6 OR #7 OR #8
#16 #9 OR #10 OR #11 OR #12 OR #13
#17 #14 AND #15 AND 16
