# Supplementary material for: Tocilizumab as monotherapy or combination therapy for treating active rheumatoid arthritis: a meta-analysis of efficacy and safety reported in randomized controlled trials
Source: Arthritis Res Ther. 2016 Sep 22;18:211. doi: 10.1186/s13075-016-1108-9 (PMC5034420; doi:10.1186/s13075-016-1108-9)
Supplement: Additional file 2: — Forest plots of American College of Rheumatology 20 (a), 50 (b), and 70 (c) responses. Meta-analyses of ACR responses for the following treatment-control combinations: (1) TCZMONO vs. TCZCOMBI; (2) TCZMONO vs. csDMARD; and (3) TCZCOMBI vs. csDMARD. (DOCX 31 kb) [file 13075_2016_1108_MOESM2_ESM.docx]

Additional file 2.

a.

I. **TCZ_COMBI_** vs. **TCZ_MONO_**

II. **TCZ_COMBI_** vs. **csDMARD**

III. **TCZ_MONO_** vs. **csDMARD**

b.

I. **TCZ_COMBI_** vs. **TCZ_MONO_**

II. **TCZ_COMBI_** vs. **csDMARD**

III. **TCZ_MONO_** vs. **csDMARD**

c.

I. **TCZ_COMBI_** vs. **TCZ_MONO_**

II. **TCZ_COMBI_** vs. **csDMARD**

III. **TCZ_MONO_** vs. **csDMARD**
