# Supplementary material for: Tocilizumab as monotherapy or combination therapy for treating active rheumatoid arthritis: a meta-analysis of efficacy and safety reported in randomized controlled trials
Source: Arthritis Res Ther. 2016 Sep 22;18:211. doi: 10.1186/s13075-016-1108-9 (PMC5034420; doi:10.1186/s13075-016-1108-9)
Supplement: Additional file 3: — Funnel plots of a DAS28 < 2.6, b ACR20, c ACR50 responses, d ACR70 responses, e AEs and f SAEs. Funnel plots of efficacy and safety outcomes for the following treatment-control combinations: (1) TCZMONO vs. TCZCOMBI; (2) TCZMONO vs. csDMARD; and (3) TCZCOMBI vs. csDMARD. (DOCX 63 kb) [file 13075_2016_1108_MOESM3_ESM.docx]

Additional file 3.

I. **TCZ** **_COMBI_** vs. **TCZ_MONO_** II. **TCZ_COMBI_** vs. **csDMARD** III. **TCZ_MONO_** vs. **csDMARD**

**f.**

**e.**

**d.**

**c.**

**a.**

**b.**
