# Supplementary material for: Tocilizumab as monotherapy or combination therapy for treating active rheumatoid arthritis: a meta-analysis of efficacy and safety reported in randomized controlled trials
Source: Arthritis Res Ther. 2016 Sep 22;18:211. doi: 10.1186/s13075-016-1108-9 (PMC5034420; doi:10.1186/s13075-016-1108-9)
Supplement: Additional file 4: — Variance in heterogeneity per meta-analysis. Table showing the heterogeneity between studies of DAS28 < 2.6, ACR 20/50/70 responses, AEs and SAEs. (DOCX 17 kb) [file 13075_2016_1108_MOESM4_ESM.docx]

Additional file 4.

|  | Meta-analysis | |  | Sensitivity analyses† | |
| --- | --- | --- | --- | --- | --- |
| Outcome measures | I^2^ (%) | P-value |  | I^2^ (%) | P-value |
| **TCZ_COMBI_** vs*.* **TCZ_MONO_** | | |  |  |  |
| DAS28 <2.6 | 0 | 0.67 |  | 0 | 0.91 |
| ACR20 | 0 | 0.89 |  | 0 | 0.92 |
| ACR50 | 0 | 0.76 |  | 0 | 0.73 |
| ACR70 | 48 | 0.10 |  | 13 | 0.33 |
| AE | 73 | 0.005 |  | 89 | 0.002 |
| SAE | 0 | 0.68 |  | 10 | 0.29 |
| **TCZ_COMBI_** vs*.* **csDMARD** | | |  |  |  |
| DAS28 <2.6 | 85 | <0.001 |  | 87 | <0.001 |
| ACR20 | 95 | <0.001 |  | 96 | <0.001 |
| ACR50 | 93 | <0.001 |  | 94 | <0.001 |
| ACR70 | 91 | <0.001 |  | 92 | <0.001 |
| AE | 33 | 0.17 |  | 24 | 0.26 |
| SAE | 18 | 0.29 |  | 26 | 0.25 |
| **TCZ_MONO_** vs*.* **csDMARD** | | |  |  |  |
| DAS28 <2.6 | 76 | 0.002 |  | 81 | 0.001 |
| ACR20 | 93 | <0.001 |  | 94 | <0.001 |
| ACR50 | 90 | <0.001 |  | 93 | <0.001 |
| ACR70 | 85 | <0.001 |  | 88 | <0.001 |
| AE | 43 | 0.14 |  | 79 | 0.03 |
| SAE | 0 | 0.94 |  | 0 | 0.97 |

TCZ = Tocilizumab; csDMARD = conventional synthetic disease modifying anti-rheumatic drugs; DAS28 remission: Disease Activity Score in 28 joint <2.6; ACR = American College of Rheumatology; AE = Adverse Events; SAE = Serious Adverse Events; † The CHARISMA study was excluded in all meta-analyses; the FUNCTION study was excluded in all meta-analyses but only in the safety outcome (AE and SAE) measures; the SAMURAI study was excluded (only in the safety meta-analyses) in the meta-analyses of TCZ_MONO_ vs. csDMARD; the SURPRISE study was excluded (only in the safety meta-analyses) in the meta-analyses of the TCZ_COMBI_ vs. TCZ_MONO_ meta-analyses.
