# Supplementary figures and images for: Tocilizumab as monotherapy or combination therapy for treating active rheumatoid arthritis: a meta-analysis of efficacy and safety reported in randomized controlled trials
Source: Arthritis Res Ther. 2016 Sep 22;18:211. doi: 10.1186/s13075-016-1108-9 (PMC5034420; doi:10.1186/s13075-016-1108-9)

Additional file 5.

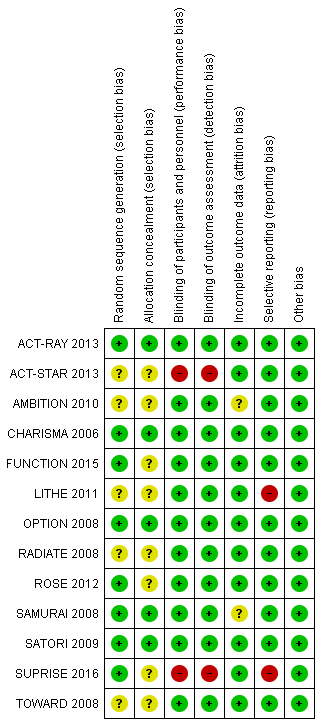

Supplement: Additional file 5: — Assessment of quality of studies (+ indicates low risk of bias, − indicates high risk of bias and ? indicates the risk of bias is unclear). Table showing the risk of bias for each study included in this meta-analyses. (DOCX 30 kb) [file 13075_2016_1108_MOESM5_ESM.docx]
